# Hsa_circ_0004872 mitigates proliferation, metastasis and immune escape of meningioma cells by suppressing PD-L1

**DOI:** 10.1007/s11011-024-01345-4

**Published:** 2024-05-21

**Authors:** Kuo Chen, Zhengming Huang, Changsheng Liu, Qian Ouyang, Qing Yan, Wei Zheng, Yongkai Huang

**Affiliations:** 1 Neurosurgery Department, Zhuzhou Hospital affiliated to Xiangya Medical College of Central South University, No.116, South Changjiang Road, Tianyuan District, 412000 Zhuzhou City, Hunan Province People’s Republic of China; 2 School of Automation, Central South University, 410083 Changsha, Hunan Province People’s Republic of China; 3 Graduate Collaborative Training Base of Zhuzhou Central Hospital, Hengyang Medical School, University of South China, Hengyang, Hunan Province People’s Republic of China

**Keywords:** hsa_circ_0004872, Meningiomas, EIF4A3, PD-L1, Immune escape

## Abstract

Meningioma is a prevalent intracranial malignancy known for its aggressive growth. Circular RNAs (circRNAs) play a crucial role in the development of various cancers. However, their involvement in meningioma remains understudied. This study aimed to investigate the function and underlying mechanism of hsa_circ_0004872 in meningioma. The molecular expression of hsa_circ_0004872, PD-L1 and EIF4A3 was identified by RT-qPCR and/or western blot assays. Cell viability, migration, and invasion were assessed through CCK-8 and Transwell assays, respectively. Cytotoxicity was determined using an LDH assay, and cell apoptosis was monitored by flow cytometry. The RNA and protein interactions were assessed through RNA-protein immunoprecipitation (RIP) and RNA pull down analyses. Our findings revealed that hsa_circ_0004872 expression was significantly downregulated in both meningioma tissue samples and cells. Overexpression of hsa_circ_0004872 inhibited the proliferation, metastasis, and immune escape of meningioma cells, as well as enhanced the cytotoxicity of CD8+ T cells by suppressing PD-L1. Furthermore, hsa_circ_0004872 directly interacted with EIF4A3, leading to the degradation of PD-L1 mRNA. Finally, inhibiting EIF4A3 improved the proliferation, metastasis, and immune escape of meningioma cells, as well as the cytotoxicity of CD8+ T cells. Our study demonstrated that hsa_circ_0004872 mitigated the proliferation, metastasis,and immune escape of meningioma cells by targeting the EIF4A3/PD-L1 axis. These findings suggested that hsa_circ_0004872 and EIF4A3 might serve as promising biological markers and therapeutic targets for meningioma treatment.

## Highlights

Hsa_circ_0004872 was downregulated in meningiomas and negatively correlated with PD-L1.

Overexpression of hsa_circ_0004872 hindered the metastasis and immune escape of meningioma cells.

Hsa_circ_0004872 competitively interacted to EIF4A3 to decay PD-L1 mRNA.

EIF4A3 upregulation facilitated the metastasis and immune escape of meningioma cells.

## Introduction

Meningiomas, accounting for over 30% of intracranial tumors, are considered the most common primary central nervous system tumors. It was estimated that the incidence rate was nearly 7.8 cases per 100,000 people (Ostrom et al. 2017). Meningiomas are tumors that originate from arachnoid cells that belonged to the dura’s inner surface, and their occurrence could be found on the intracranial surface or in extracranial organs, leading to neurological deficits through the compression of brain regions or cranial nerves (Maggio et al. 2021). The grades of meningiomas could be classified into three different stages (benign, atypical and anaplastic) based on histomorphology, which includes mitotic activity and brain invasion (Pawloski et al. 2021). The recommended treatment, according to guidelines from the European Association of Neuro-Oncology, involves surgery with adjuvant radiotherapy (Nowosielski et al. 2017). However, these treatment modalities have limitations in reducing recurrence and improving overall survival (Rogers et al. 2018). Considering these challenges, exploring the molecular mechanisms underlying meningioma progression represents a promising avenue for advancing diagnosis and treatment.

Immune checkpoints play a critical role in regulating interactions between immune cells and tumor cells. Programmed death-ligand 1 (PD-L1) is one of the most critical checkpoints (Dyck and Mills 2017). It is a transmembrane protein whose expression serves as a reliable predictor for the efficacy of checkpoint inhibitor therapies in various cancers (Ju et al. 2020; Zhang et al. 2004). A recent study revealed that PD-L1 expression was highest in anaplastic grade among three grades of meningiomas, indicating the vital role of PD-L1 in meningiomas (Karimi et al. 2021). Eukaryotic translation initiation factor 4A3 (EIF4A3) belongs to the RNA-binding protein family (Sakellariou and Frankel 2021), and has been reported to be related to the tumorigenesis of glioblastoma (Wei et al. 2021). Using bioinformatic tools, we have identified potential binding interactions between PD-L1 and EIF4A3. To the best of our knowledge, the interaction and roles of EIF4A3 and PD-L1 have not been studied in meningiomas.

Circular RNAs (circRNAs) are highly conserved RNA molecules formed by covalently closed loops, arising from unconventional splicing of mRNA precursors (Patop et al. 2019). Aberrant circRNA expression has been documented in various cancers, suggesting their significant role in tumorigenesis and development (Jiang et al. 2021b). Growing evidence indicated that circRNAs could be involved in the regulation of cancer malignant biological phenotypes through diverse molecular mechanisms (Chen et al. 2022). For example, Huang et al. reported that hsa_circRNA_104348 functions as a competitive endogenous RNA (ceRNA) to regulate miR-187-3p/RTKN2 signaling, thus contributing to hepatocellular cancer tumorigenesis and lung metastasis (Zheng et al. 2020). Furthermore, the aberrantly expressed circURI1 in gastric cancer could directly recruit hnRNPM to regulate alternative splicing of genes, participating in the malignant proliferation and metastasis of gastric cancer cells (Wang et al. 2021). RNA-seq analysis conducted by Chen et al. revealed that circRHOBTB3 was down-regulated in colorectal cancer, gain-of function experiments showed that circRHOBTB3 directly interacted with HuR and promoted its ubiquitination degradation, thus reducing the stability of PTBP1 mRNA to restrain tumor metastasis (Chen et al. 2021).However, the role of circRNAs in meningiomas remains largely unexplored.

In previous research, hsa_circ_0004872, originating from exons 2 ~ 4 of the host MAPK1 gene, was found to be significantly downregulated in meningiomas. Notably, hsa_circ_0004872 has been identified as a tumor-suppressing factor in gastric cancer progression (Jiang et al. 2021a; Ma et al. 2020), suggesting its potential significance in meningioma development. Predictive analysis using circInteractome software has revealed putative binding sites between hsa_circ_0004872 and EIF4A3. Therefore, we hypothesized that hsa_circ_0004872 could target the EIF4A3/PD-L1 axis to regulate the proliferation, metastasis, and immune escape of meningioma cells.

## Methods

### Tissue samples

The clinical study received approval from the Ethics Committee of Zhuzhou Hospital, affiliated with Xiangya Medical College of Central South University. Written informed consent was obtained from all participating individuals. Meningioma tissue samples and non-cancerous tissues (*n* = 35) were collected from patients meeting the following selection criteria: (i) a diagnosis of meningioma with no history of other tumors, and (ii) the availability of complete clinical information, including age, gender, and tumor volume. The collection and utilization of these tissue samples adhered to the National Regulations on the Use of Clinical Samples in China.

### Cell culture and transfection

Normal meningothelial cells (MECs) and meningioma cell lines IOMM-Lee and CH157-MN were obtained from the American Type Culture Collection (Manassas, VA, USA). The CD8^+^ T cells that isolated form adult male peripheral blood was purchased from Bluefcell (#BFN60810741, Shanghai, China). All these cell types were cultured in Dulbecco’s Modified Eagle’s Medium (Thermo Fisher Scientific, Carlsbad, CA, USA) supplemented with 10% fetal bovine serum (Sigma, St. Louis, MO, USA) in a 5% CO_2_ humidified atmosphere at 37 °C. For stimulation of CD8^+^ T cells, the cells were incubated with Dynabeads® that coated with anti-CD3 and anti-CD28 (#11161D, Thermo Fisher Scientific, Carlsbad, CA, USA) for two days, thus the activated CD8^+^ T cells were co-cultured with meningioma cells by a transwell chamber.

For the construction of knockdown or overexpression cellular models, pcDNA3.1-circ_0004872, pcDNA3.1-PD-L1, sh-EIF4A3, as well as their respective negative controls (pcDNA3.1 and shNC), were sourced from GenScript (Shanghai, China). Cell transfections were performed using Lipofectamine 2000 (Invitrogen, Carlsbad, CA, USA), following the manufacturer’s instructions.

### Cell viability

Cell viability was assessed using a CCK8 assay kit (Solarbio, Beijing, China) according to the manufacturer’s protocols. Briefly, cells were seeded in 96-well plates at a density of 5 × 10^3^ cells per well and incubated under various conditions for 24, 48, and 72 h. Subsequently, CCK8 solution (10 µL) was added to each well and maintained for 2 h at 37 °C in the dark. A microplate reader (Molecular Devices, Sunnyvale, CA, USA) was used to measure absorbance at 450 nm.

### Cell migration and invasion assays

To assess the metastasis of meningioma cells, chambers (8.0 μm, Corning, NY, USA) covered with/without Matrigel (BD Biosciences, Bedford, MA, USA) were used. Cells were harvested after the indicated treatment and then incubated in serum-free medium for 6 h. Subsequently, cells were collected and resuspended in serum-free medium. Afterwards, 200 µL of cell suspension (5 × 10^4^ cells) was plated in the upper chambers, with 600 µL of complete medium in the bottom chamber. After 48 h of culture, migratory and invasive cells were fixed with 4% paraformaldehyde and stained with 0.5% crystal violet. The cells were counted using a light microscope (Olympus, Tokyo, Japan). Notably, invasion experiments involved the use of Matrigel in the upper chamber.

### LDH assay

Cytotoxicity was determined using the Cytotoxicity Detection Kit (LDH) (Beyotime, Shanghai, China) according to the manufacturer’s instructions. IOMM-Lee and CH157-MN cells subjected to different treatments were co-cultured with CD8^+^ T cells at varying ratios of 2:1, 3:1, and 5:1 for 48 h. A six-well transwell system (0.4 mm pore size membrane; Corning, Oneonta, NY, YSA) was used to assess the interactions between CD8^+^ cell and meningiomas cells. After co-culture, CD8 + T cells were harvested for apoptosis detection, and meningiomas cells were obtained for LDH release assay. Cytotoxicity of CD8^+^ T cells against meningioma cells was quantified through the LDH assay. The difference in absorbance (OD_490_-OD_680_) represented LDH activity, and the percentage cytotoxicity was calculated using the formula: % cytotoxicity = [1-(OD _case_-OD _effector cell_)/OD _target cell_] × 100%.

### Cell apoptosis

IOMM-Lee and CH157-MN cells with different treatment were co-cultured with CD8^+^ T cells at dissimilar ratios of 2:1, 3:1 and 5:1 for 48 h. Then, the harvested co-cultured CD8 + T cells as described in LDH assay was used for apoptosis measurement. Briefly, the transfected cells were trypsinized, rinsed with cold PBS buffer, and then resuspended at a density of 5 × 10^5^ cells/mL. Then, cells were stained with 5 µL of Annexin V-FITC/PI staining solution (BD Biosciences, San Jose, CA, USA) for 15 min. After washing with 1× binding buffer, the cells were treated with 10 µL of PI (Solarbio, Beijing, China) as recommended by the manufacturer. The apoptosis rate was quantified using a flow cytometer (BD Biosciences, San Jose, CA, USA).

### RT-qPCR

Total RNA was extracted using TRIzol reagent (Invitrogen, Carlsbad, CA, USA). Following the protocols of the reverse transcription reagent kit (TaKaRa, Tokyo, Japan), cDNA was synthesized. Real-time quantitative PCR (RT-qPCR) was performed using SYBR Green qPCR Mix (TaKaRa) on the ABI quantitative PCR system (Waltham, MA, USA). β-actin served as an endogenous control, and relative RNA expression was calculated using the 2^−ΔΔCt^ method. The gene primers used were as follows:

- hsa_circ_0004872-F:5’-CCCGTGTTGCAGATCCAGAC -3’;
- hsa_circ_0004872-R:5’- GGGTTCTCTGGCAGTAGGTC-3’;
- PD-L1-F: 5’- ACCACCACCAATTCCAAGAG-3’;
- PD-L1-R: 5’- GATGGCTCCCAGAATTACCA -3’;
- EIF4A3-F: 5’- GACTCTGGAAGGCATCAAGC-3’;
- EIF4A3-R: 5’- AGTGAAGTTGGCTTCCCTCA-3’;
- β-actin-F: 5’- CCCTGGAGAAGAGCTACGAG -3’;
- β-actin-R: 5’- CGTACAGGTCTTTGCGGATG-3’.

### Western blot analysis

Total proteins were extracted using RIPA lysis buffer (Bocai, Shanghai, China). After centrifugation, the supernatant was collected, and protein concentrations were determined using a BCA Assay Kit (Beyotime, Shanghai, China). Proteins were separated by 10% SDS-PAGE gel. Following electrophoresis, proteins were transferred to PVDF membranes (Millipore, MA, USA) and blocked with 5% non-fat milk. Membranes were then incubated with primary antibodies, including anti-PD-L1 (ab205921, abcam, Cambridge, MA, USA), anti-EIF4A3 (ab180573, abcam), and anti-GAPDH (ab8245, abcam) at 4℃ for overnight. On the following day, the membranes were probed with an HRP-conjugated rabbit anti-mouse IgG secondary antibody (ab6728, abcam) for 2 h at room temperature. An ECL kit (Invitrogen) was used to develop the protein bands, and the band density was quantified using ImageJ software.

### RIP assay

The RNA-protein immunoprecipitation (RIP) assay was conducted using the EZ-Magna RIP Kit (Millipore, Bellerica, MA). Cell lysates were extracted and incubated with anti-EIF4A3 (ab180573, abcam) or anti-IgG antibody (ab172730, abcam) that coated with protein A sepharose beads at 4 °C overnight. Samples were subsequently treated with proteinase K, and the extracted RNAs were analyzed using RT-qPCR.

### RNA pull down

Magnetic RNA-protein Pulldown Kit (Thermo Scientific, Waltham, MA, USA) was obtained for RNA pull-down analysis. Briefly, the biotin-labeled probes (biotin-sense, biotin-antisense) that targeted the junction site of hsa_circ_0004872 were synthesized by GenePharma (Shanghai, China). The antisense sequence of hsa_circ_0004872 was used as a non-specific control. To obtain cell lysates, cells were treated with lysis buffer containing protease and RNase inhibitors and incubated on ice for 30 min. Subsequently, the biotin-labeled probes were incubated with streptavidin magnetic beads at 4 °C overnight. The obtained lysates were incubated with prepared magnetic beads for overnight. Then, the beads were collected and the proteins pulled down by probes-beads complexes were harvested using a protein elution buffer. Finally, the probe interacted proteins were determined using western blot.

### RNA stability

In brief, a total of 2 mg/ml actinomycin D was used for cell treatment. Total RNA was extracted at various time points (0, 3, 6, 9, and 12 h), and the relative RNA levels were determined by RT-qPCR to evaluate RNA stability.

### Statistical analysis

Quantitative data were presented as the mean ± standard deviation (SD). All statistical analyses were conducted using GraphPad Prism 7.0. A Student’s t-test was employed for comparisons between two groups, while one-way analysis of variance followed by Tukey’s post hoc test was used for comparisons involving three or more groups. *P* < 0.05 was considered statistically significant. All experiments were performed in triplicate.

## Results

### Hsa_circ_0004872 was downregulated and PD-L1 was upregulated in meningiomas

**Fig. 1:**
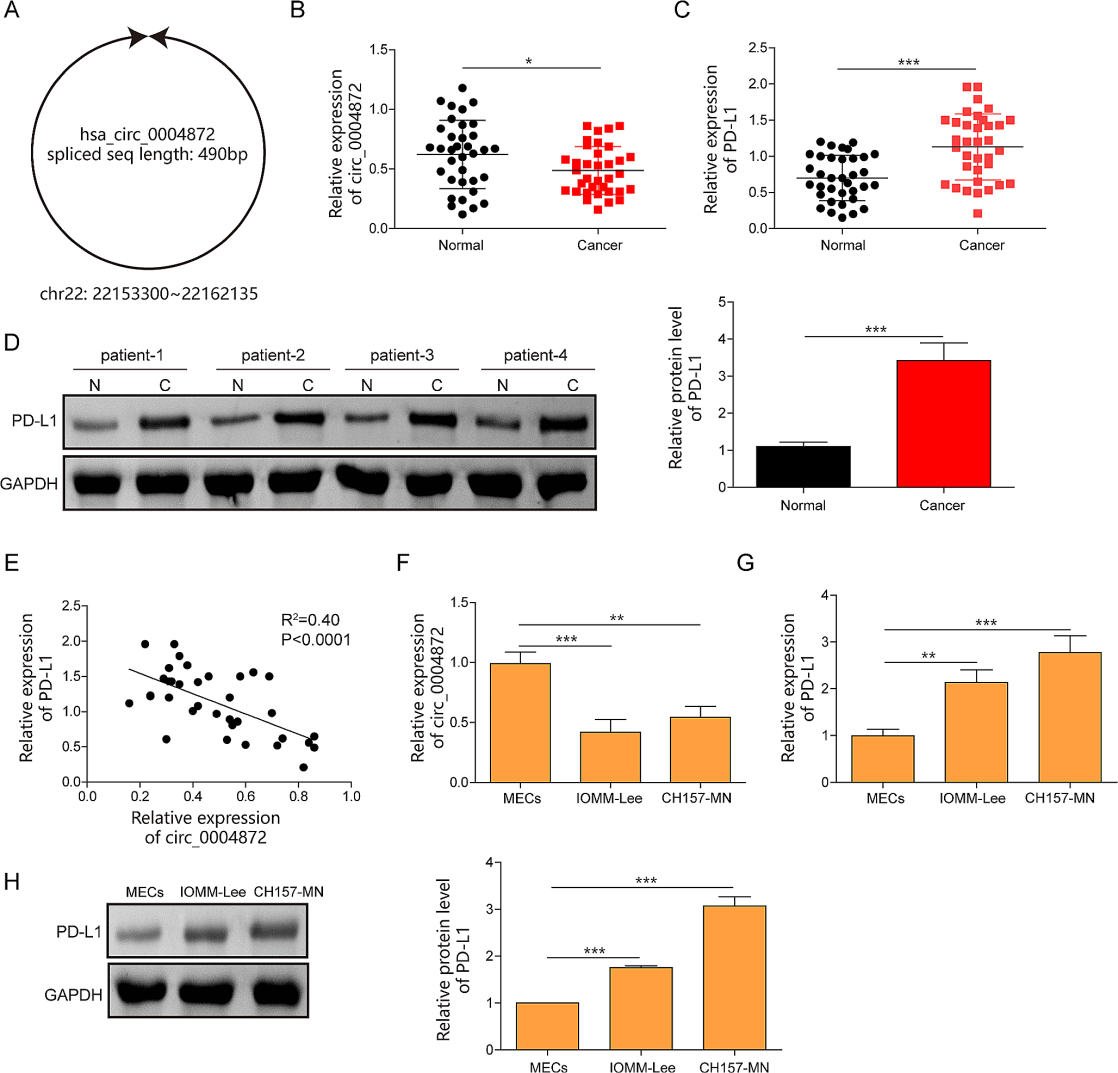
Hsa_circ_0004872 was downregulated and PD-L1 was upregulated in meningiomas. **A** The schematic diagram of the hsa_circ_0004872. **B** The expression of hsa_circ_0004872 in meningiomas tissues was determined by RT-qPCR (*n *= 35). **C** The mRNA expression of PD-L1 in meningiomas tissues was evaluated by RT-qPCR (*n* = 35). **D **The protein expression of PD-L1 was tested by western blot (*n* = 35). **E** The correlation between hsa_circ_0004872 and PD-L1 analyzed by Pearson correlation analysis. **F**,** G** The expression of hsa_circ_0004872 and PD-L1 were determined by RT-qPCR in meningiomas cells (IOMM‐ Lee, CH157-MN) and MEC cells. **H** The protein of PDL1 in meningiomas cells (IOMM‐ Lee, CH157-MN) and MEC cells was quantified by western blot. *P < 0.05. **P < 0.01

Hsa_circ_0004872 is located at chr22: 22,153,300–22,162,135 and is derived from the NM_002745 transcript (gene symbol: MAPK1), with a full length of 490 bp. The schematic diagram of hsa_circ_0004872 was presented in Fig. 1A. Clinical experiments revealed a significant downregulation of hsa_circ_0004872 and an overexpression of PD-L1 in meningioma tissues compared to that in non-cancerous tissues (Fig. 1B and D). Pearson correlation analysis showed a negative correlation between hsa_circ_0004872 and PD-L1 in patients with meningiomas (Fig. 1E). Furthermore, RT-qPCR assay presented that hsa_circ_0004872 level was decreased in meningioma cell lines IOMM-Lee and CH157-MN compared to normal MECs (Fig. 1F). Consistently, both mRNA and protein expression of PD-L1 were also elevated in meningioma cells (Fig. 1G and H).

### Overexpression of hsa_circ_0004872 inhibited proliferation, migration, and invasion of meningioma cells through suppressing PD-L1

**Fig. 2:**
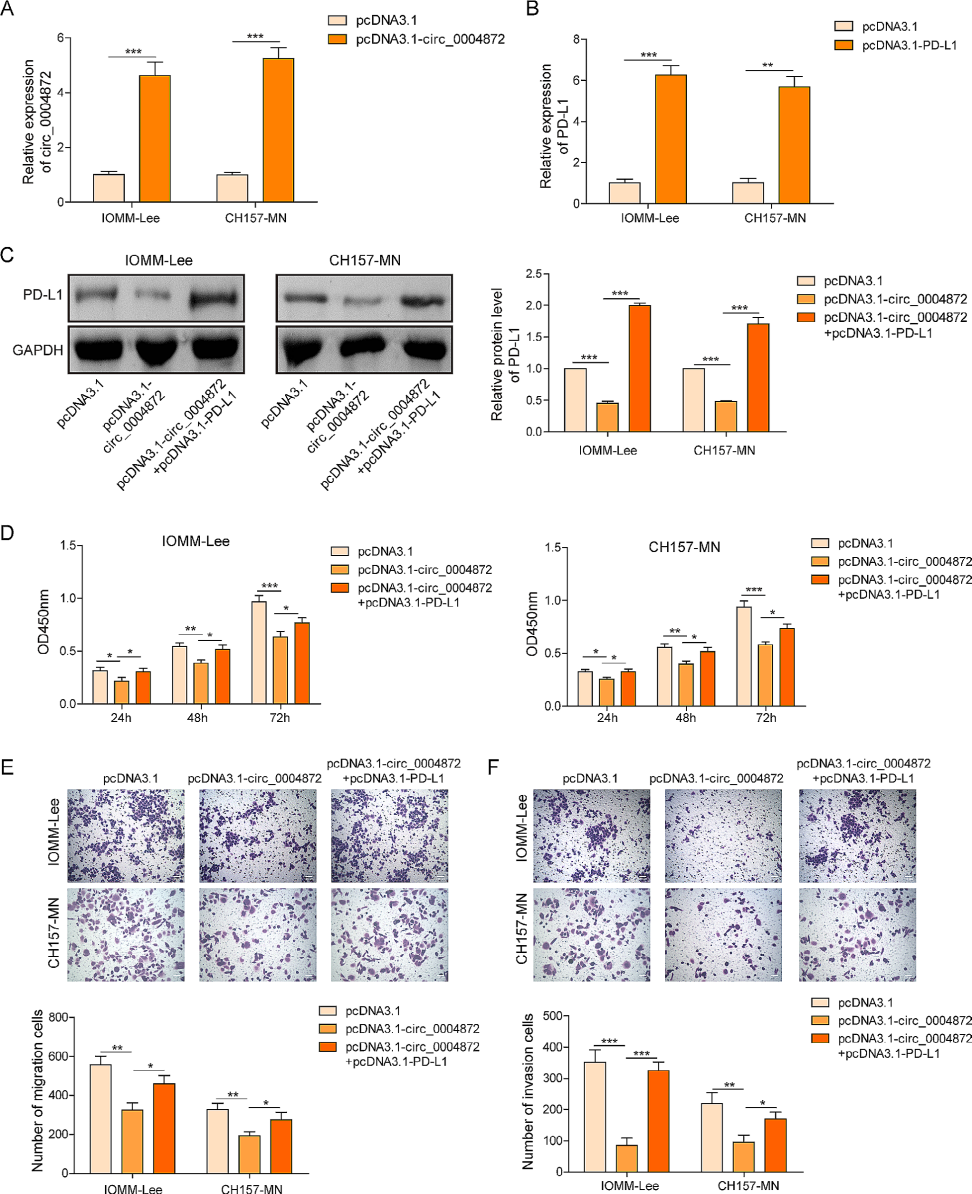
Overexpression of hsa_circ_0004872 inhibited proliferation, migration, and invasion of meningiomas cells through suppressing PD-L1. **A** The expression of hsa_circ_0004872 in IOMM‐ Lee and CH157-MN cells with pcDNA3.1 or pcDNA3.1-circ transfection was evaluated by RT-qPCR. **B**, **C** The mRNA and protein expression of PD-L1 in IOMM‐ Lee and CH157-MN cells with pcDNA3.1 or pcDNA3.1-PD-L1 transfection was determined by RT-qPCR and western blot. **D** Cell viability of IOMM‐ Lee and CH157-MN cells was detected by CCK8 assay after hsa_circ_0004872 overexpression with/without PD-L1 cooverexpression. **E**, **F** Cell migration and invasion measured using Transwell assay upon hsa_circ_0004872 overexpression with/without PD-L1 co-overexpression. *P < 0.05. **P < 0.01

Building upon the findings above, we aimed to elucidate the regulatory roles of hsa_circ_0004872 and PD-L1 in meningiomas. First, overexpressing cellular models for hsa_circ_0004872 and PD-L1 were constructed and validated by RT-qPCR. The results demonstrated an increase in hsa_circ_0004872 expression after transfection with pcDNA3.1-circ plasmids (Fig. 2A) and an elevation in PD-L1 mRNA level in the presence of pcDNA3.1-PD-L1 (Fig. 2B). Western blot analysis further showed that the overexpression of hsa_circ_0004872 significantly reduced PD-L1 levels. Conversely, overexpression of PD-L1 reversed these effects in both IOMM-Lee and CH157-MN cells (Fig. 2C). CCK-8 assay showed that hsa_circ_0004872 overexpression led to reduced cell viability at 24, 48, and 72 h, while PD-L1 overexpression counteracted the inhibitory effects of hsa_circ_0004872 by promoting cell proliferation (Fig. 2D). Transwell assay provided additional evidences that hsa_circ_0004872 overexpression inhibited cell migration and invasion, however, transfection of pcDNA3.1-PD-L1 markedly reversed these effects (Fig. 2E, F). These findings collectively suggested that hsa_circ_0004872 overexpression restrained the cell proliferation, migration, and invasion in vitro.

### Overexpression of hsa_circ_0004872 repressed the immune escape in meningioma cells

**Fig. 3:**
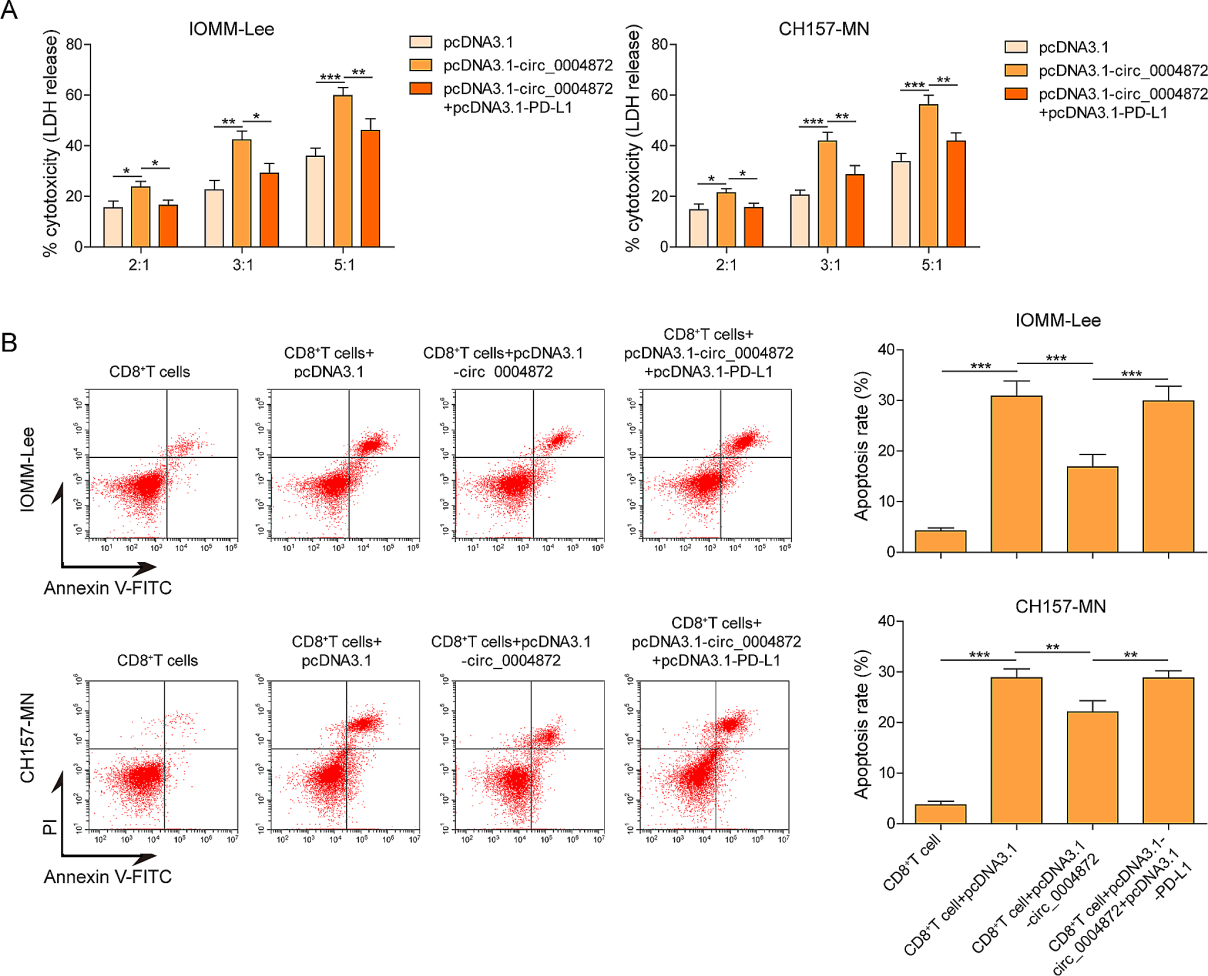
Overexpression of hsa_circ_0004872 repressed the immune escape of meningiomas cells. After indicated treatment, IOMM‐ Lee and CH157-MN cells were co-cultured with CD8+ T cells at dissimilar ratios of 2:1, 3:1 and 5:1 for 48h. Then, **A** the cytotoxicity of CD8+ T cells against meningioma cells were quantified via LDH assay. **B** The cell apoptosis of CD8+ T cells was monitored using flow cytometry. *P < 0.05. **P < 0.01

The transfected IOMM-Lee and CH157-MN cells were then co-cultured with CD8^+^ T cells. To assess the cytotoxicity of CD8^+^ T cells against meningioma cells, LDH release assay were conducted. As shown in Fig. 3A, overexpression of hsa_circ_0004872 enhanced the cytotoxicity of CD8^+^ T cells against meningioma cells, while this effect was hindered when PD-L1 was co-overexpressed. Furthermore, flow cytometry analysis revealed an increase in the apoptosis rate of CD8^+^ T cells after co-culturing with meningioma cells. Notably, hsa_circ_0004872 overexpression reduced the percentage of apoptotic CD8^+^T cells, however, the apoptosis rate of CD8^+^T cells was further elevated upon PD-L1 overexpression (Fig. 3B).

### The binding relations between hsa_circ_0004872 and EIF43A

**Fig. 4:**
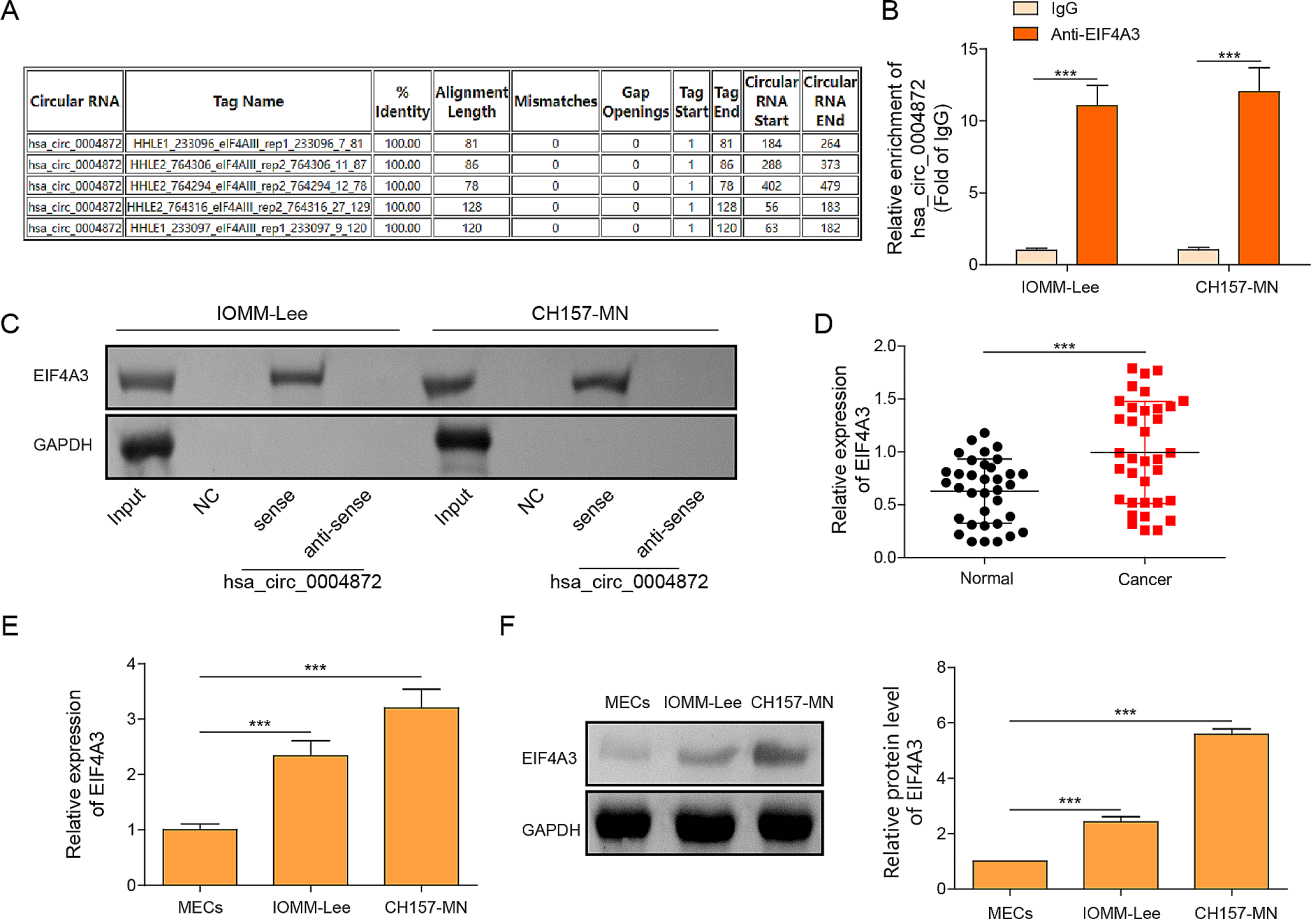
The binding relations between hsa_circ_0004872 and EIF43A. **A** The predicted binding region between hsa_circ_0004872 and EIF43A using Circinteractome database (https://circinteractome.nia.nih.gov/index.html). **B** RIP assay proved the interaction between hsa_circ_0004872 and EIF43A. **C** RNA pull down assay demonstrated the interaction between hsa_circ_0004872 and EIF43A. **D** The expression of EIF4A3 in meningiomas tissues was determined by RT-qPCR. **E**, **F** The level of EIF4A3 in meningiomas cells (IOMM‐ Lee, CH157-MN) and MEC cells was determined by RT-qPCR and western blot. *P < 0.05. **P < 0.01

Using the bioinformatic tool circinteractome, potential binding interactions between EIF4A3 and hsa_circ_0004872 were identified (Fig. 4A). Subsequently, RIP analysis showed a significant abundance of hsa_circ_0004872 in the anti-EIF4A3 group compared to the anti-IgG group (Fig. 4B). The RNA pull-down assay also revealed that biotin-labeled hsa_circ_0004872 notably pulled down the EIF4A3 protein, indicating a direct interaction between hsa_circ_0004872 and EIF4A3 (Fig. 4C). Furthermore, RT-qPCR assay suggested the higher EIF4A3 mRNA expression in meningioma tissues than that in non-cancerous tissues (Fig. 4D). Consistently, both mRNA and protein levels of EIF4A3 were elevated in IOMM-Lee and CH157-MN cells compared to normal MECs (Fig. 4E and F). These findings indicated a direct interaction between hsa_circ_0004872 and EIF4A3.

### Overexpression of hsa_circ_0004872 blocks interactions between EIF4A3 and PD-L1 mRNA

**Fig. 5:**
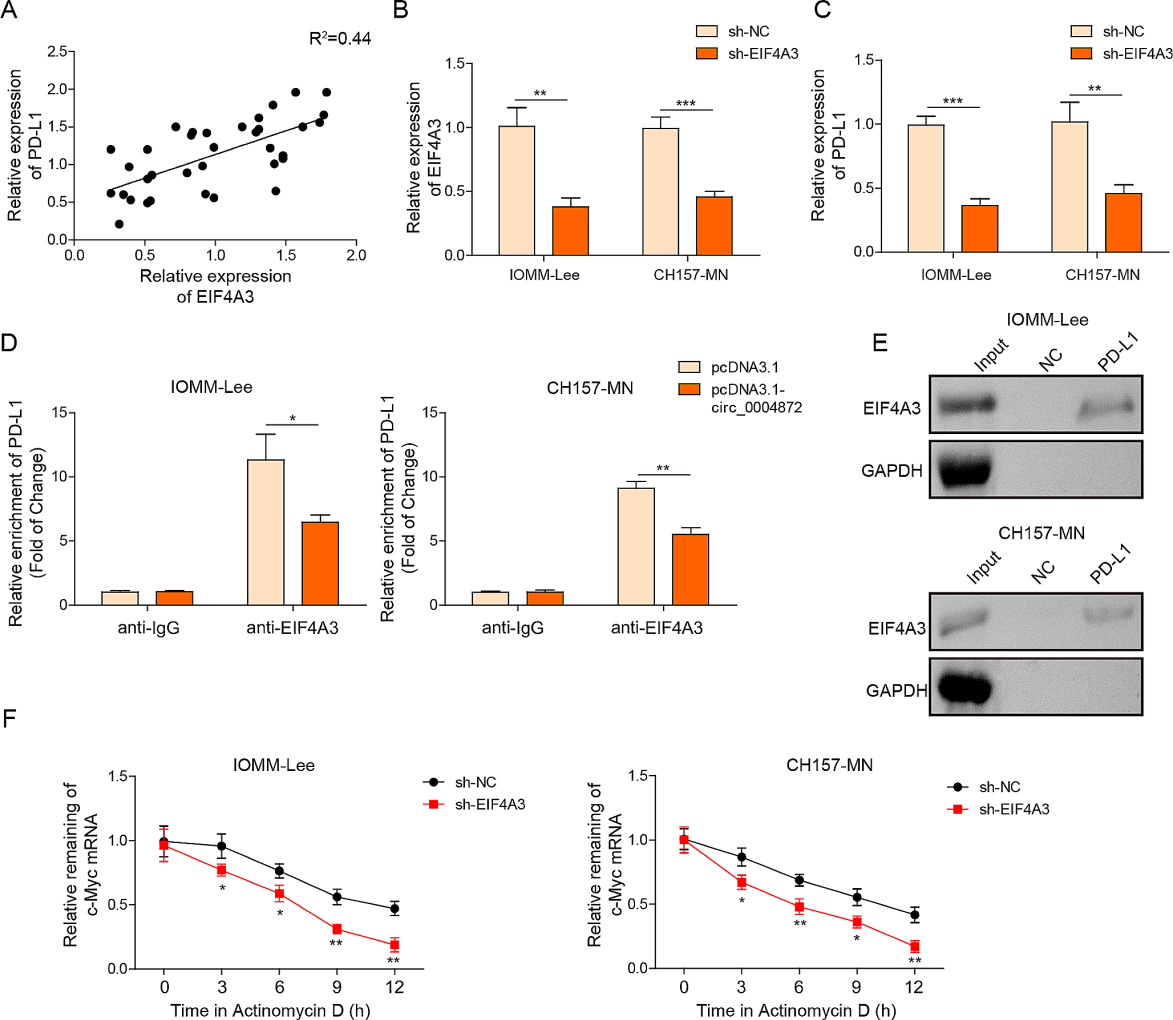
Overexpression of hsa_circ_0004872 blocked the interactions between EIF4A3 and PDL1 mRNA. **A** The correlation between hsa_circ_0004872 and EIF43A analyzed by Pearson correlation analysis. **B**, **C** The mRNA levels of EIF43A and PD-L1 in IOMM‐ Lee and CH157-MN cells with shNC or shEIF4A3 transfection were determined by RT-qPCR. **D** RIP assay proved the interaction between PD-L1 and EIF43A in IOMM‐ Lee and CH157-MN cells with pcDNA3.1 or pcDNA3.1-circ transfection. **E** RNA pull down assay demonstrated the interaction between PD-L1 and EIF43A. **F** The RNA stability of PD-L1 mRNA after actinomycin D was identified using RT-qPCR. *P < 0.05. **P < 0.01

Pearson correlation analysis showed a positive association between PD-L1 and EIF4A3 (Fig. 5A). To investigate the molecular association between EIF4A3 and PD-L1, shEIF4A3 was designed and transfected into IOMM-Lee and CH157-MN cells to silence EIF4A3 expression. RT-qPCR experiment revealed that sh-EIF4A3 transfection markedly repressed the mRNA levels of PD-L1 and EIF4A3 (Fig. 5B and C). Subsequently, the RIP assay confirmed that EIF4A3 directly targeted PD-L1 mRNA, while ectopic expression of hsa_circ_0004872 inhibited the interaction between EIF4A3 and PD-L1 mRNA (Fig. 5D). Additionally, RNA pull-down analysis showed that biotin-labeled PD-L1 notably pulled down the EIF4A3 protein (Fig. 5E). Moreover, after actinomycin D treatment, the mRNA stability of PD-L1 mRNA was significantly repressed when EIF4A3 was silenced (Fig. 5F). These findings collectively indicated that hsa_circ_0004872 and PD-L1 competitively bound to EIF4A3 in meningioma cells.

### EIF4A3 exhibited an oncogenic role in metastasis and immune escape of meningioma cells

**Fig. 6:**
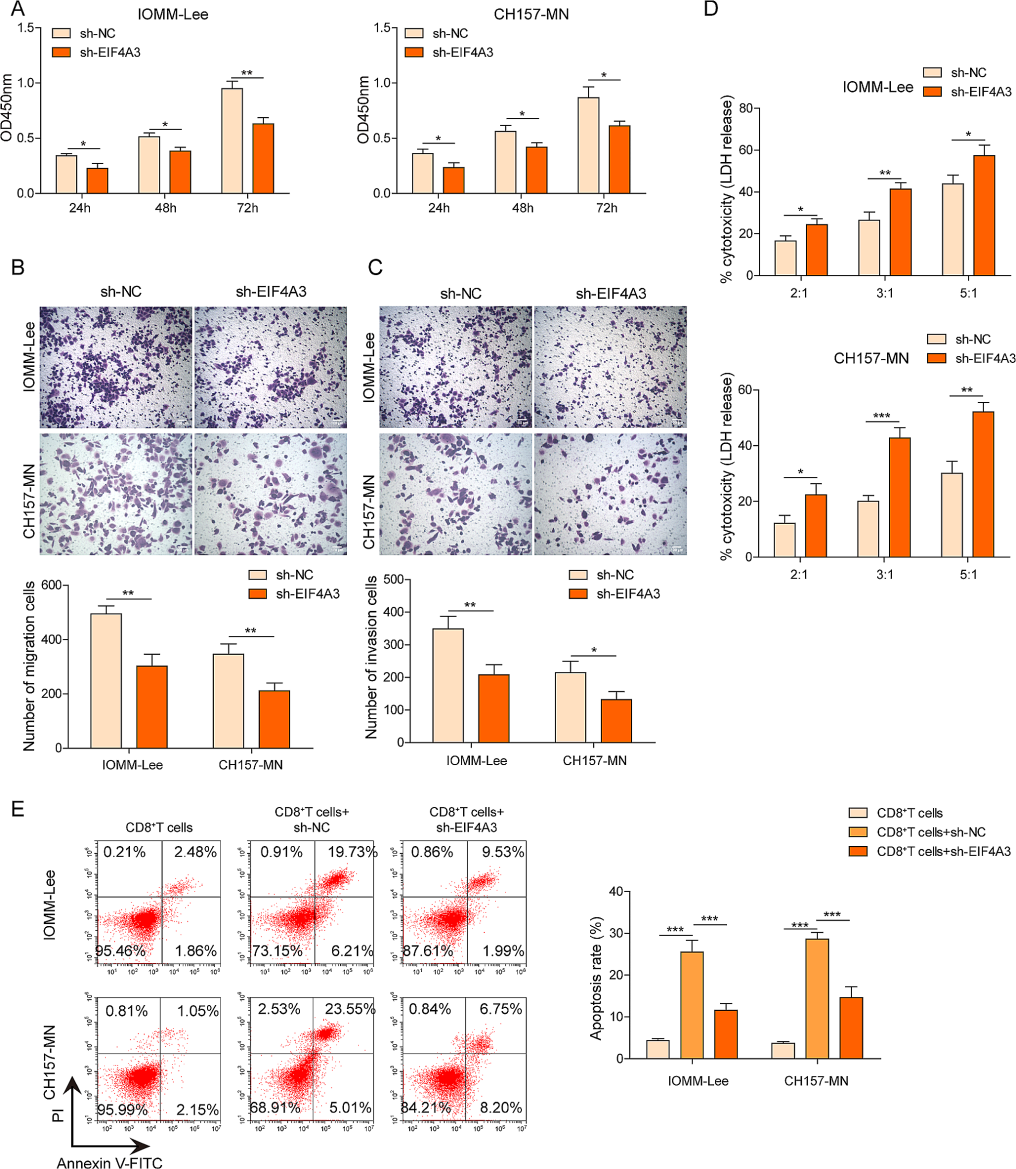
EIF4A3 exhibited an oncogenic role in metastasis and immune escape of meningiomas cells. IOMM‐ Lee and CH157-MN cells were transfected with shNC and shEIF4A3, respectively. **A** Cell viability detected by CCK8 assay. **B**, **C** Cell migration and invasion measured using Transwell assay. The transfected IOMM‐ Lee and CH157-MN cells were co-cultured with CD8+ T cells at dissimilar ratios of 2:1, 3:1 and 5:1 for 48h. Then, **D** the cytotoxicity of CD8+ T cells against meningioma cells were quantified via LDH assay. **E** The cell apoptosis of CD8+ T cells was monitored using flow cytometry. *P < 0.05. **P < 0.01

In this section, we investigated the detailed roles of EIF4A3 in meningioma. CCK8 results demonstrated that the loss-of-function of EIF4A3 significantly decreased cell viability in both IOMM-Lee and CH157-MN cells at 24, 48, and 72 h (Fig. 6A). Transwell assay confirmed that the depletion of EIF4A3 notably inhibited cell migration and invasion compared to the shNC group (Fig. 6B and C). A co-culture system was established between meningioma cells and CD8^+^ T cells. LDH assay revealed that knockdown of EIF4A3 promoted LDH production in meningioma cells, suggesting that the cytotoxicity of CD8^+^ T cells was enhanced (Fig. 6D). Meanwhile, flow cytometry assay showed that the apoptosis rate of CD8^+^ T cells was elevated after EIF4A3 silencing (Fig. 6E). These data indicated that the inhibition of EIF4A3 ameliorated metastasis and immune escape in meningioma cells.

## Discussion

The tumor microenvironment (TME) represents the biological milieu surrounding tumors, typically comprised of endothelial cells, immune cells, fibroblasts, and signaling molecules, all of which play pivotal roles in tumor development (Spill et al. 2016). Recent studies have emphasized the recruitment and activation of the host immune system as a significant factor in regulating cancer progression, with cytotoxic CD8^+^ memory T cells playing a crucial role in improving cancer prognosis by recognizing specific antigens on cancer cells and facilitating their elimination (Arneth 2019). The PD-L1/PD-1 signaling pathway has emerged as a vital participant in tumor-associated immunosuppression, affecting the activation of T lymphocytes and resulting in enhanced immune tolerance and evasion (Jiang et al. 2019). Notably, PD-L1 expression has been found to be elevated in meningioma tissues and served as an independent prognostic marker for meningiomas (Karimi et al. 2020). Han et al. have proposed that PD-L1 might play a critical regulatory role in the aggressive phenotype of higher-grade meningiomas (Han et al. 2016). Furthermore, high-grade meningiomas usually harbor an immunosuppressive TME, and their aggressive abilities were closely associated to the high levels of PD-L1 and regulatory T (Treg) cells (Du et al. 2015). Studies have demonstrated that PD-L1 regulated various biological traits of cancer cells, including metastasis, migration, and invasion. For instance, Zhang et al. reported that PD-L1 contributed to promoting the metastasis of pancreatic cancer cells (Zhang et al. 2020). Additionally, PD-L1 has been shown to enhance colorectal cancer cell migration ability through the RAS/MEK/ERK signaling pathway (Cao et al. 2022). Nevertheless, the roles and implications of the PD-L1 immune checkpoint in meningiomas remain a topic of debate. In the present study, we have demonstrated an upregulation of PD-L1 expression in meningiomas and cells, and discovered a novel molecule, hsa_circ_0004872, which exhibited a significant negative correlation with PD-L1. This finding suggested that hsa_circ_0004872 might play a role in regulating immunity and metastasis in meningiomas.

CircRNAs are single-stranded nucleotides with a covalently closed structure and have been reported to be widely expressed in viruses, eukaryotes, and mammals (Capel et al. 1993). Their unique structure imparts circRNAs with a longer half-life and higher stability compared to linear RNAs. Recently, there has been a growing body of research highlighting the involvement of circRNAs in various biological processes related to the development of cancer and other diseases (Ghafouri-Fard et al. 2021). For instance, Chen et al. discovered that circRNA_0000285 was significantly upregulated in cervical cancer, and its knockdown inhibited cell growth and metastasis in tumor cells (Chen et al. 2019). Similarly, hsa_circRNA_104348 was found to facilitate proliferation, migration, and invasion while suppressing apoptosis in hepatocellular carcinoma cells (Huang et al. 2020). Regarding cicrMAPK1, the previous reported that circMAPK1 was responsible for tumor suppressor gene encoding, which inhibited the MAPK1 pathway and suppressed the proliferation of gastric cancer cells (Lu et al. 2021). In addition, it also revealed that circMAPK1 could sponge miR-22-3p and promote the proliferation and migration of vascular smooth muscle cells (Fu et al. 2021). In our study, we observed the abnormal downregulation of hsa_circ_0004872 in meningiomas and conducted functional investigations. Our recent results confirmed that the gain-of-function of hsa_circ_0004872 significantly enhanced the malignant proliferation, migration, and invasion capabilities of meningioma cells in vitro. Moreover, hsa_circ_0004872 also enhanced the cytotoxic effects of CD8^+^ T cells against meningioma cells and reduced the apoptosis of CD8^+^ T cells. Notably, all these biological effects of hsa_circ_0004872 could be markedly attenuated by co-overexpression of PD-L1. Based on these findings, we interred that hsa_circ_0004872 exerted as a suppressor in meningiomas by decreasing the metastasis, immune escape and immunosuppressive TME via downregulation PD-L1.

CircRNAs can interact with RNA-binding proteins and serve as scaffolds and recruiters in various molecular processes. For example, Liang et al. reported that circDCUN1D4 interacted to human antigen R and thioredoxin-interacting protein mRNA, forming a stable RNA-protein ternary complex that led to the suppressing of lung cancer cells metastasis (Liang et al. 2021). Likewise, in the present work, our results demonstrated that EIF4A3 could directly bind to hsa_circ_0004872 and PD-L1. Notably, the enforced hsa_circ_0004872 remarkably blocked the interaction between EIF4A3 and PD-L1, suggesting a regulatory relationship among hsa_circ_0004872, EIF4A3, and PD-L1. A previous study by Song et al. revealed that increased EIF4A3 expression promoted PD-L1 activation, further supporting our findings (Song et al. 2020). Wang et al. also found that hsa_cir_0068631 recruited EIF4A3 to maintain c-Myc mRNA stability, thus mediating breast cancer progression (Wang et al. 2021). Furthermore, EIF4A3 has been shown to inhibit circ_0087429 expression by binding to its flanking regions, leading to enhanced proliferation, migration, invasion, and angiogenesis in cervical cancer (Yang et al. 2022). In a similar vein, Liu et al. suggested that EIF4A3-induced circTOLLIP facilitated the proliferation and metastasis of hepatocellular carcinoma cells through the miR-516a-5p/PBX3/EMT axis (Liu et al. 2022). All these reports underscored the oncogenic roles of EIF4A3 in tumorigenesis. As expected, our experiments, for the first time, discovered that EIF4A3 was significantly upregulated in meningioma tissues and cells, and had a positive correlation with PD-L1. Loss-of-function experiments revealed that silencing EIF4A3 markedly repressed the proliferation, metastasis, and immune escape of meningioma cells in vitro, and promoted the immunosuppressive TME formation.

In conclusion, our study revealed that hsa_circ_0004872 plays a pivotal role in mitigating the proliferation, metastasis, and immune escape of meningioma cells through its interaction with the EIF4A3/PD-L1 axis. These findings provided promising molecular targets for the development of clinical therapeutic strategies for meningiomas. However, to further substantiate these findings, additional investigations involving animal models and clinical trials are warranted. Additionally, exploring other relevant biological pathways will be crucial to obtain a comprehensive understanding of the underlying mechanisms.

## Data Availability

All data generated or analyzed during this study are available on request to the corresponding author.
